# A triboelectric nanogenerator based on cosmetic fixing powder for mechanical energy harvesting

**DOI:** 10.1038/s41378-019-0066-1

**Published:** 2019-07-01

**Authors:** Kequan Xia, Yue Chi, Jiangming Fu, Zhiyuan Zhu, Hongze Zhang, Chaolin Du, Zhiwei Xu

**Affiliations:** 1 Ocean College, Zhejiang University, 316021 Zhejiang, China; 2 Nanjing Electronic Devices Institute, 210016 Jiangsu, China

**Keywords:** Electrical and electronic engineering, Electronic properties and materials

## Abstract

In this work, we use commercial powder particulates (a cosmetic fixing powder) as triboelectric materials for constructing a triboelectric nanogenerator (CFP-TENG). Through finger pressing, the CFP-TENG generated approximate open-circuit voltage, short-circuit current, and maximum power density values of 1141 V, 521 µA, and 570.96 μW/cm^2^, respectively. Thirty-seven commercial blue LEDs can be easily lit up by the CFP-TENG. Moreover, this TENG, which was designed as a novel palette structure for harvesting mechanical energy from bicycle motion, serves as a self-powered bicycle speed sensor. In addition, the cosmetic fixing powder can be used as an effective material to enhance the triboelectric property of skin. This study provides an effective method for developing a cost-effective TENG without the use of complex surface micro-/nano-texturing.

## Nanogenerators: Harvesting energy with cosmetic powder

Cosmetic powder acts as an effective component in triboelectric nanogenerators (TENGs) for producing electricity from mechanical movements. The finding, by Zhiyuan Zhu, Zhiwei Xu of Zhejiang University and colleagues in China, is a step towards large-scale production. TENGs generate electricity from small movements, such as finger motions or from raindrops. Friction between their tiny electrodes causes a transfer of electrons that turns into an electrical potential when they separate. More effective, low-cost materials are needed to improve their fabrication. The team found that finger-pressing a TENG incorporating a layer of cosmetic powder can generate enough electricity to light up 37 commercial LEDs. The TENG also harvested energy from cycling. Adding a layer of the powder enhanced the natural triboelectric property of skin. This could help in the development of wearable electronics.

## Introduction

The global energy crisis is becoming increasingly severe with the fast-paced growth of the global economy^1–3^. Moreover, with the continued acceleration of human civilization, exhaustion of fossil energy in the first half of the twenty-first century is expected^4,5^. Renewable energy for electricity generation has been considered a means of solving the energy crisis^6–10^. Solar energy, tidal energy, mechanical motion, and thermal changes are all considered potential forms of energy that are convertible into electrical energy in the environment. Among these, mechanical energy is the most extensively distributed type, as it occurs in diverse forms^11,12^. Daily activities such as walking, running, cycling, and even tiny facial expressions (for example, smiling and crying) are accompanied by mechanical distortions, suggesting that the human body constitutes a pivotal means of mechanical power generation^13,14^. However, it is difficult to apply traditional electromagnetic induction generators for harvesting mechanical energy from the surrounding environment (human daily activities, etc.).

In 2012, Prof. Zhonglin Wang (Georgia Institute of Technology) proposed the first-ever triboelectric nanogenerator (TENG), which converts mechanical energy from the surrounding environment into electrical energy on the basis of the triboelectric effect^15–25^. During the past few years, the TENG has received considerable attention and has been widely applied to multidisciplinary fields owing to its outstanding output performance, sustainable power output, and ease of integration^26–34^. Several materials (for example, PDMS, Kapton, Al, and Teflon, each with its own merits and demerits) have been applied to the construction of TENGs^35–41^. Although silica-based powder is also used as one of the triboelectric pair^42^, the output voltage is relatively low (~11 V). The triboelectric substances proposed for TENG construction should (ideally) be low cost, yield rapidly self-forming films, be processable without the need for cumbersome micro-/nano-processing, and (if possible) be readily available.

In this work, we propose a novel concept of a powder TENG that uses powder particulates (for example, cosmetic fixing powder) as triboelectric materials for fabricating a triboelectric nanogenerator (CFP-TENG). This fabrication, which is realized without the use of surface micro-/nano-texturing techniques that rely on expensive equipment and complex technology, may drive the large-scale application of TENGs in multidisciplinary fields. In our work, this powder and Teflon tape were used as the triboelectric pair. The CFP-TENG can, through finger pressing, produce approximate open-circuit voltage, short-circuit current, and power density values of 1141 V, 521 µA, and 570.96 μW/cm^2^, respectively. Thirty-seven commercial blue LEDs, which were assembled into the word “ZJU”, could be lit up by the CFP-TENG. In addition, this TENG, which was designed as a novel palette structure to harvest mechanical energy from bicycle motion, serves as a self-powered speed sensor. In addition, the cosmetic fixing powder can be used as an effective material to enhance the triboelectric property of skin, which is significant for the development of a human-based TENG.

## Results

**Fig. 1:**
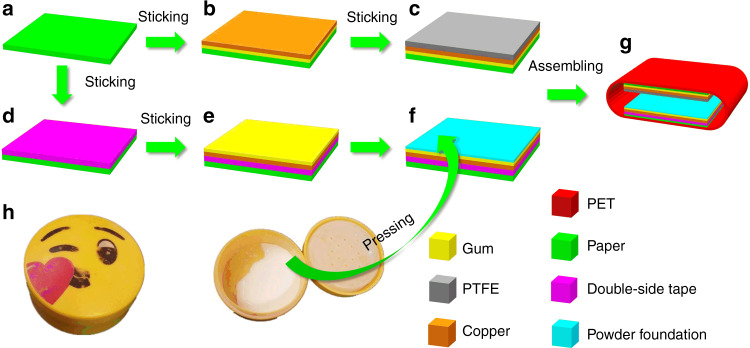
**Preparation process of the CFP-TENG**

The design and fabrication process of the CFP-TENG device is schematically presented in Fig. 1. First, a sheet of paper was cut into two pieces of paper substrate (size: 3 cm × 3 cm), as shown in Fig. 1a. A piece of copper tape (3 cm × 3 cm) was then pasted onto the paper surface (see Fig. 1b). Subsequently, Teflon tape was pasted onto the surface of the copper tape, thereby forming the top section of the TENG, as shown in Fig. 1c. Another paper substrate was obtained by pasting the double-sided tape onto the paper substrate surface, as shown in Fig. 1d, and then attaching the copper to the surface of the tape. Afterward, the cosmetic fixing powder was applied onto the glue side of the copper tape. Excess powder was then removed by air blowing, thereby forming the bottom section of the TENG (see Fig. 1e, f). A piece of Polyethylene terephthalate (PET) film served as the supporting structure for assembly of the TENG, as illustrated in Fig. 1g.

**Fig. 2:**
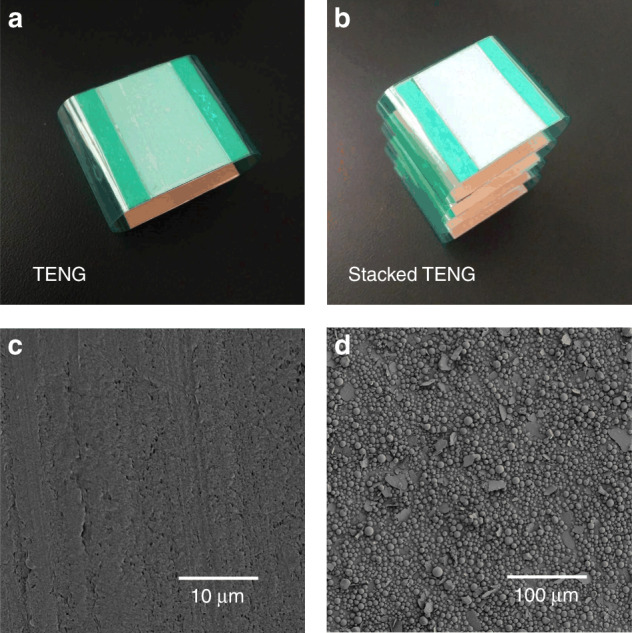
**Images of the device and triboelectronic surfaces.** Photographs of the **a** CFP-TENG unit and **b** stacked CFP-TENG. SEM image of the **c** Teflon tape surface and **d** cosmetic fixing powder layer surface

Figure 2a, b shows photographs of the CFP-TENG (one unit) and the stacked CFP-TENG. Representative scanning electron microscopy images showing the surface of the Teflon tape and cosmetic fixing powder layer are presented in Fig. 2c, d. A mechanical vibrator was used to activate the CFP-TENG. The top and bottom of the TENG were affixed to the pressing surface of the vibrator and a flat panel, respectively. The respective electrical output signals were then measured with a digital oscilloscope.

**Fig. 3:**
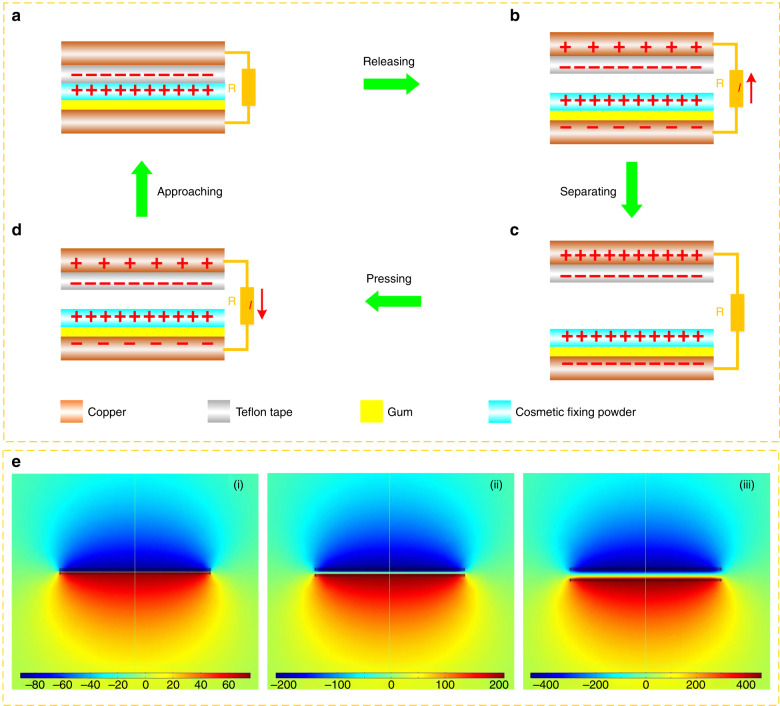
**Schematic and modeling analysis of the device. a**–**d** Working principle of the CFP-TENG. **e** Numerical calculations (as assessed with the help of COMSOL) of the potential distribution across TENG electrodes, at each step (i–iii), subjected to open-circuit conditions (the voltage unit is volts)

The working principle of the CFP-TENG is schematically illustrated in Fig. 3. When Teflon tape is in contact with cosmetic fixing powder, electron transfer from the powder layer to the tape (Fig. 3a). A latent electrical difference is expected with separation of the tape from the powder. This type of latent difference drives the electron flow via the external loads from the top Cu foil to the bottom Cu foil. This flow screens the positive triboelectric charges available on the paper, thereby producing an output current signal (Fig. 3b). A new electrical equilibrium is expected at the largest separation in a complete contact–separation cycle (Fig. 3c). Subsequent to this equilibrium, contact between the Teflon tape and the cosmetic fixing powder layer is re-established, leading to an imbalance between the stimulated charges on the Cu electrodes. This imbalance results in electron flow back to the top Cu foil, thereby producing a reversed output current signal (Fig. 3d). When full contact between the tape and paper is re-established, the CFP-TENG reverts to its initial position, as shown in Fig. 3a. To understand this mechanism, the latent distribution is simulated with COMSOL multiphysics software. An open-circuit scenario is considered for the three positions of the one-unit CFP-TENG (see Fig. 3e).

**Fig. 4:**
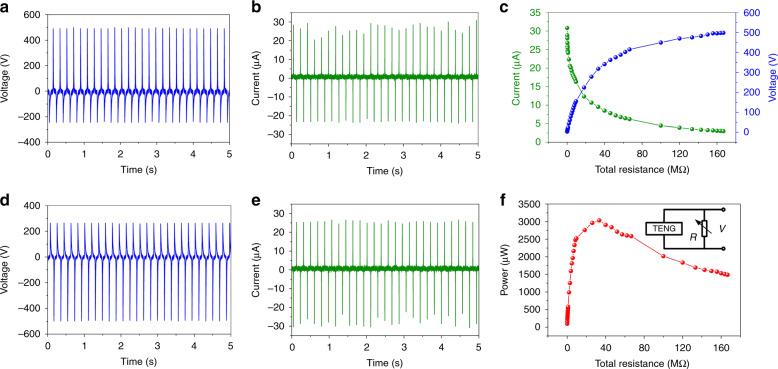
**Electrical output of the device.** Output voltage and current of the CFP-TENG calculated using the **a**, **b** forward connection and **d**, **e** reverse connection. Dependence of **c** the output voltage and current on the total resistance and **f** the power density on the total resistance

An adjustable resistor (100 kΩ to 1 GΩ) was used as the external load of the fabricated TENGs, and the electronic voltage on the resistor was measured. The output current can be derived from the measured voltage and the total resistance. The voltage (under a 1 GΩ load) and current (under a 100 kΩ load) of the fabricated CFP-TENG are 498 V and 30.8 μA, respectively (see Fig. 4a, b), when triggered by a mechanical vibrator (amplitude: ∼5 cm, frequency: 5 Hz). For the reverse connection of the oscilloscope to the CFP-TENG, the output voltage and current signals are inverted (as shown in Fig. 4d, e), which indicates that the signals were generated by the CFP-TENG. Considering the 200 MΩ probe of the oscilloscope, the total resistance can be calculated by *R*_t_ = *R* ×200 MΩ/(*R* + 200 MΩ), and the corresponding output performance of CFP-TENG is further investigated by evaluating the output power associated with the voltage and current occurring at equivalent total resistances ranging from 99.95 kΩ to 166.67 MΩ (see Fig. 4c). With increasing total resistance, the current amplitude decreases owing to a loss of resistance, whereas the voltage increases. In addition, an approximate maximum power density value of 369.17 μW/cm^2^ corresponding to a total resistance of ∼26 MΩ (see Fig. 4f) is obtained. In addition, the output performances with and without cosmetic fixing powder are compared in the Supporting Information.

**Fig. 5:**
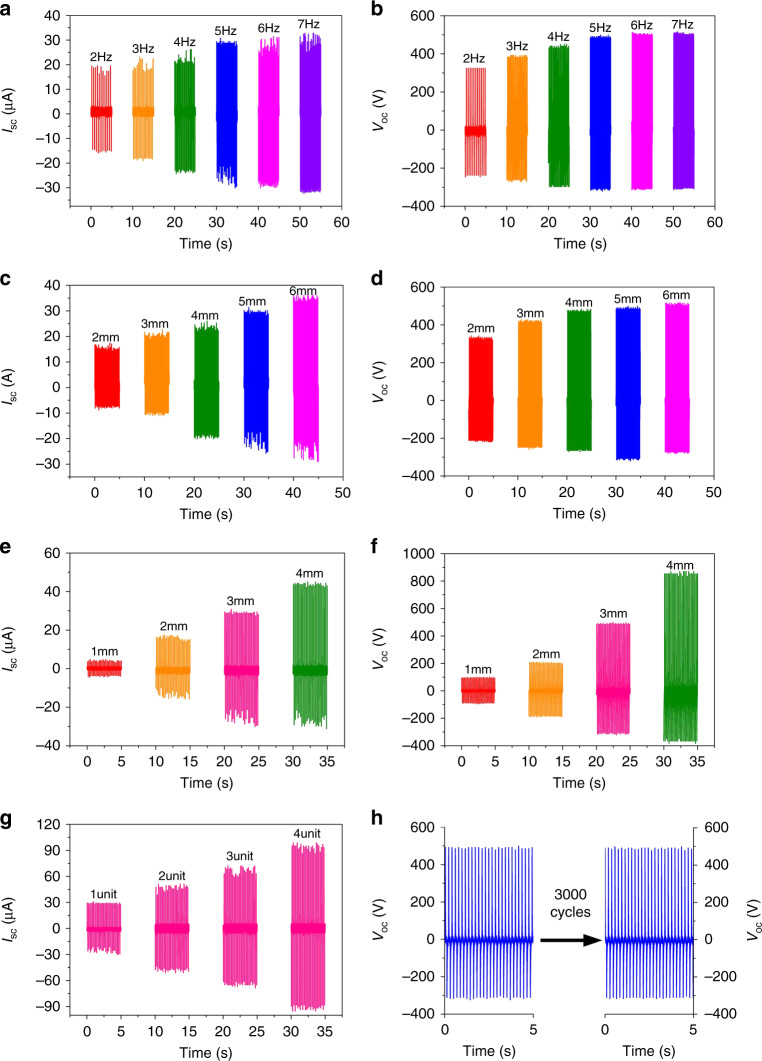
**Performance and stability of the device**. Approximate values of *V*_OC_ and *I*_SC_ at various **a**, **b** frequencies (CFP-TENG size: 3 cm × 3 cm, maximum separation distance: 5 mm), **c**, **d** separation distances (mechanical frequency: 5 Hz, CFP-TENG size: 3 cm × 3 cm), and **e**, **f** CFP-TENG sizes (working frequency: 5 Hz, separation distance of the TENG: 5 mm. **g** Output current of the CFP-TENG with various unit numbers. **h** Stability verification of the fabricated CFP-TENG via 3000 cycles of continuous operation

The electronic output performance of the CFP-TENG, based on contact separation, exhibits a high dependence on the contact frequency, separation distance, and device size. Figure 5a, b shows the dependence of the *V*_OC_ and *I*_SC_ values of CFP-TENG on contact frequency for a given device size and separation distance of 3 cm × 3 cm and 5 mm, respectively. Moreover, the output voltage increases from 325 to 515 V when the contact frequency increases from 2 to 7 Hz. Nonetheless, the *I*_SC_ increases continuously from 19.5 to 32.8 μA when the contact frequency increases from 2 to 7 Hz. The increase in the *I*_SC_ and *V*_OC_ values is attributed to the rapid induction and charge transfer resulting from the elevated frequency contact. Figure 5c, d shows the *V*_OC_ and the *I*_SC_ values of the (3 cm × 3 cm) CFP-TENG as a function of the separation distance. Furthermore, with increasing separation distance, the *V*_OC_ increases slowly and then saturates, while the *I*_SC_ increases continuously. The *V*_OC_ and *I*_SC_ values corresponding to a contact frequency and separation distance of 5 Hz and 5 mm, respectively, of TENGs with varied sizes are shown in Fig. 5e, f. As the figure shows, *V*_OC_ and *I*_SC_ increase with increasing device size. The substantial enhancement in the output is attributed to the augmented contact region. Detailed discussions of the impact of the separation displacement and frequency on the electrical performance are provided in the Supporting Information.

Furthermore, a stacked CFP-TENG is designed and manufactured to improve the output efficiency. The *I*_SC_ values of the stacked CFP-TENGs with 2, 3, and 4 units are 51, 72, and 98 μA, respectively (see Fig. 5g). The results revealed that the output current increases with increasing number of units. Similarly, the stability of the CFP-TENG is evaluated from the findings presented in Fig. 5h. In accordance with the experimental results, a stable output voltage from the CFP-TENG is realized even after a 3000-cycle external force test.

**Fig. 6:**
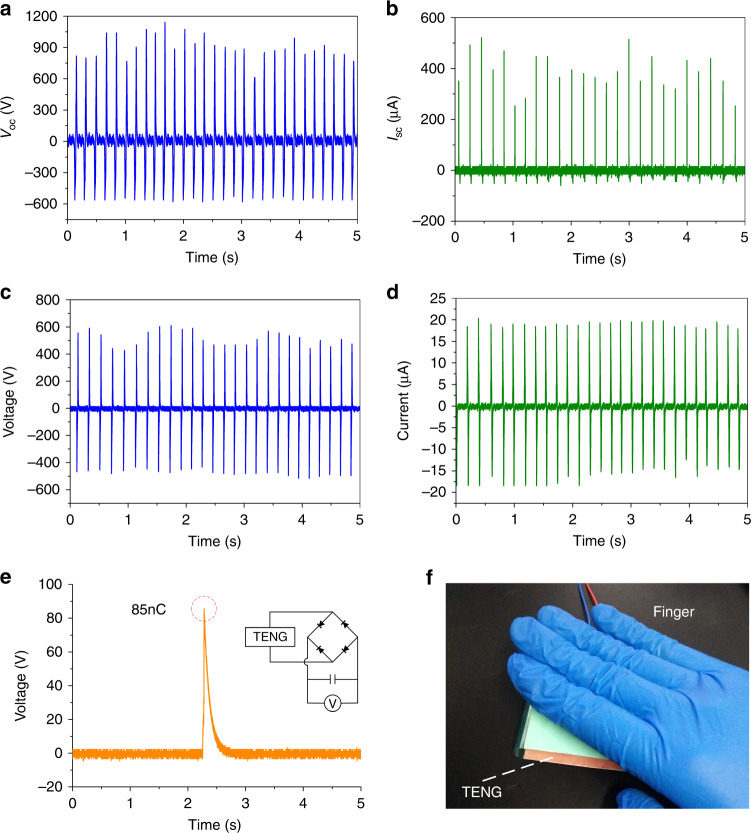
Electrical characteristics of the CFP-TENG powered by physical movement (finger pressing). Approximate **a** *V*_OC_ and **b** *I*_SC_ values of the CFP-TENG; **c** output voltage and **d** current corresponding to a match load of 30 MΩ (equivalent total resistance: 26.09 MΩ); **e** voltage of a 1 nF capacitor linked to the CFP-TENG by means of a full-wave rectifier bridge, indicating the quantity of charge transferred in one cycle; and **f** a CFP-TENG triggered by finger pressing

According to a previous study^43^, the human body produces hundreds of watts through body movements. Therefore, harvesting low-frequency power generated by human physical movements is considered a potential means of realizing self-powered wearable electronics. In this work, we demonstrate that a CFP-TENG (5 cm × 5 cm) can be activated via finger pressing (see Fig. 6f). As shown in Fig. 6a, b, *I*_SC_ and *V*_OC_ reach approximate values of 521 μA and 1141 V, respectively. When a match load of 30 MΩ (equivalent total resistance: 26.09 MΩ) is connected to the CFP-TENG, the maximum output power, corresponding to an output voltage of 610 V and current of 23.4 μA, is reached (see Fig. 6c, d). The corresponding maximum output power (12.383 mW) and power density (570.96 μW/cm^2^) are adequate for various low-energy-consumption microelectronic gadgets. We also performed tests aimed at determining the charging potential of the fabricated CFP-TENG connected to a 1 nF capacitor by means of a full-wave rectifier bridge. The results revealed that ∼85 nC of charge is transferred in one cycle.

## Discussion

**Fig. 7:**
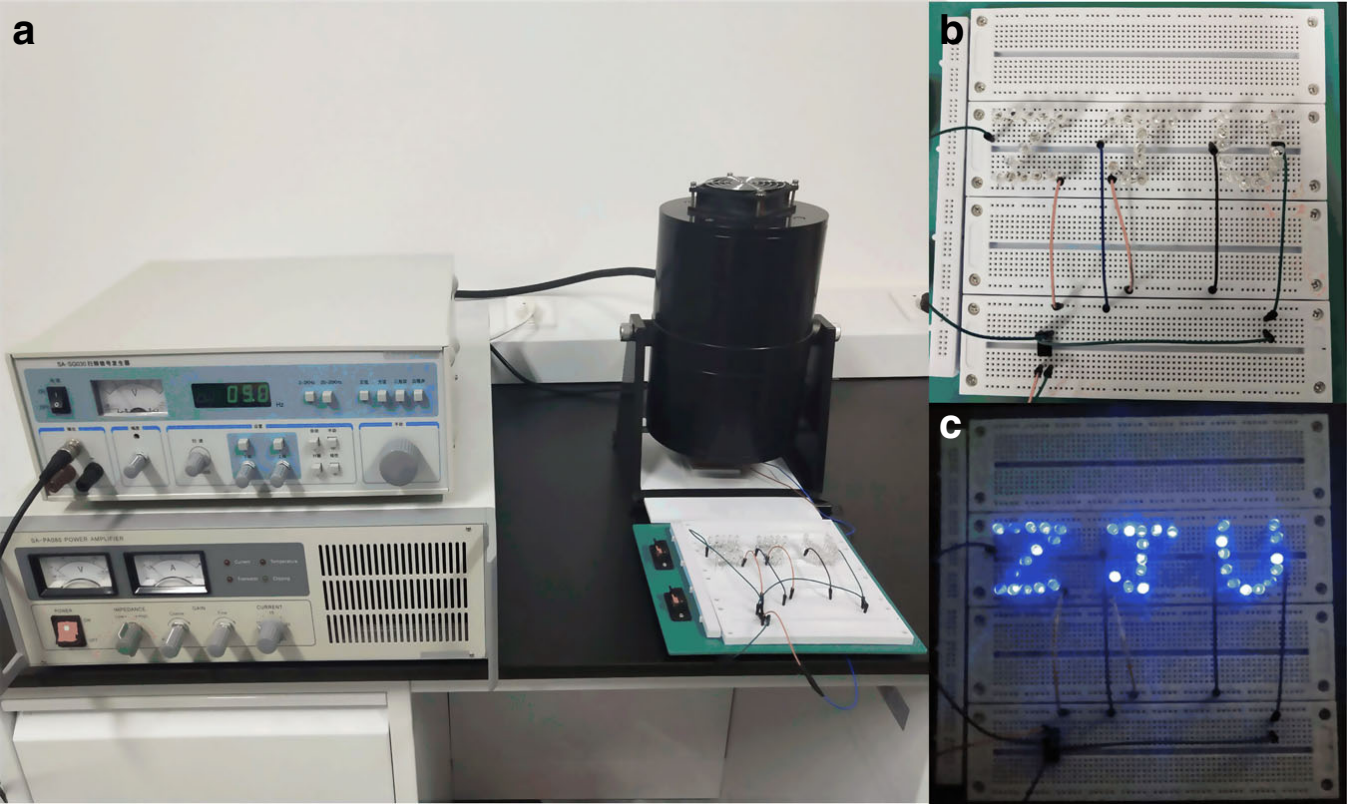
**Driving capability of the device. a** A CFP-TENG connected in series with 37 commercial blue LEDs (the inset shows a 3 cm × 3 cm TENG); **b**, **c** 37 commercial blue LEDs powered by finger pressing of the CFP-TENG

To demonstrate the potential of the CFP-TENG as an energy source, a 3 cm × 3 cm CFP-TENG was linked to 37 commercial blue LEDs in series (as presented in Fig. 7a–c). The results revealed that the assembly of these LEDs forming the word “ZJU” could be easily lit up by the TENG.

**Fig. 8:**
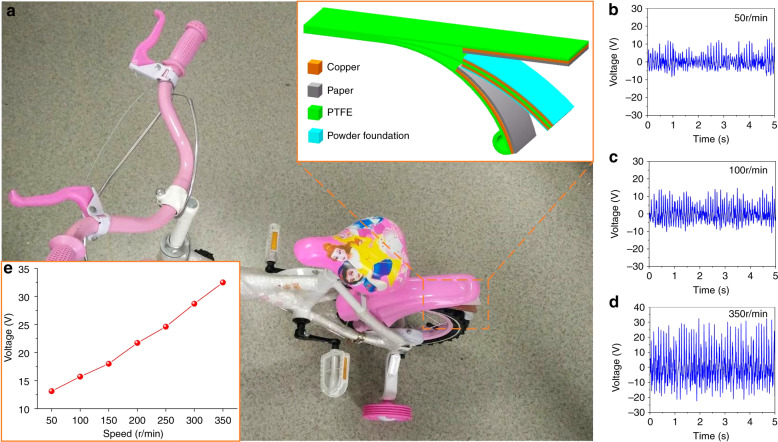
**Harvesting mechanical power associated with bicycle movement. a** Photograph of a bicycle with a TENG (inset shows the palette structure of the TENG); **b**–**d** output voltage signals corresponding to three movement states (50, 100, and 350 r.p.m.). **e** The output voltages subjected to varied speeds

In addition, we propose a novel palette structure for harvesting the mechanical power associated with bicycle movement (see Fig. 8a). Moreover, the proposed structure can also monitor the bicycle speed. The corresponding output voltage of the device (under a 1 GΩ load) can reach 13.1, 15.7, and 32.5 V for 50, 100, and 350 r.p.m., respectively, as shown in Fig. 8b–d. In addition, the output voltages subjected to different speeds are shown in Fig. 8e. It is observed that the output voltage is approximately linear to the speed.

**Fig. 9:**
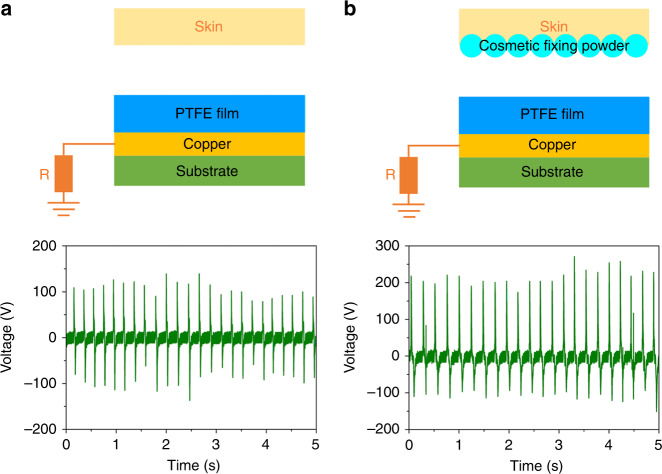
**Powder-enhanced skin-based TENG.** Schematic illustration of **a** a skin-based TENG and **b** a cosmetic fixing powder-enhanced skin-based TENG and the corresponding output performance

Furthermore, we proposed a new application of a powder-enhanced skin-based TENG. In detail, when the skin is made as the triboelectric layer, the output voltage can reach 137 V, as shown in Fig. 9a. When the skin is modified by cosmetic fixing powder, the output voltage can reach 274 V, as shown in Fig. 9b. According to the results, the cosmetic fixing powder can be used as an effective material to enhance the triboelectric property of skin, which is significant for the development of a human-based TENG.

## Conclusions

A novel concept of powder electronics that use powder particulates (a cosmetic fixing powder) as triboelectric materials for fabricating a TENG is proposed in this work. The loose powder and Teflon tape are used as the triboelectric pair. The CFP-TENG can generate approximate *V*_OC_, *I*_SC_, and maximum power density values of 1141 V, 521 µA, and 570.96 μW/cm^2^, respectively. Stable output voltage from the CFP-TENG is realized after a 3000-cycle external force test. In addition, this TENG, which was designed as a novel palette structure for harvesting mechanical energy from bicycle motion, serves as a self-powered speed sensor. Furthermore, the cosmetic fixing powder can be used as an effective material to enhance the triboelectric property of skin. This work represents a significant step towards the large-scale production of TENGs.

## Materials and methods

All materials are commercially available and used without further processing.

## Supplementary information

SUPPLEMENTAL MATERIAL

Fig. S1 (supporting information)

Fig. S2 (supporting information)
