# Supplementary material for: A triboelectric nanogenerator based on cosmetic fixing powder for mechanical energy harvesting
Source: Microsyst Nanoeng. 2019 Jul 1;5:26. doi: 10.1038/s41378-019-0066-1 (PMC6799839; doi:10.1038/s41378-019-0066-1)
Supplement: Supplementary file 1 — SUPPLEMENTAL MATERIAL [file 41378_2019_66_MOESM1_ESM.docx]

Supporting Information

**A triboelectric nanogenerator based on cosmetic fixing powder for mechanical energy harvesting**

Kequan Xia^1^, Yue Chi^1^, Jiangming Fu^1^, Zhiyuan Zhu^1*^, Hongze Zhang^2^, Chaolin Du^1^, Zhiwei Xu^1*^

^1^Ocean College, Zhejiang University, 316021, Zhejiang, China;

^2^Nanjing Electronic Devices Institute, 210016, Jiangsu, China

*Corresponding author: Zhiyuan Zhu, Email: [zyzhu@zju.edu.cn](mailto:zyzhu@zju.edu.cn)

Zhiwei Xu, Email: xuzw@zju.edu.cn

**S.1 The output performances with and without cosmetic fixing powder**

In this study, the output performances with and without cosmetic fixing powder are compared. Specifically, we designed and fabricated two TENGs using PTFE-gum and a PTFE-cosmetic fixing powder triboelectric pair (with the same size and shape), respectively, as shown in Fig. S1(a1), (a2). The output current and voltage with an external load of 100 KΩ and 1 GΩ were defined as the I_sc_ and V_oc_, respectively. According to the experimental results presented in Fig. S1(b, c), the I_sc_ and V_oc_ of the gum-based TENG reached 8.7 μA and 151 V, whereas the I_sc_ and V_oc_ of the CFP-TENG reached 19.5 μA and 325V. Therefore, the powder TENG can increase the output performance.

**
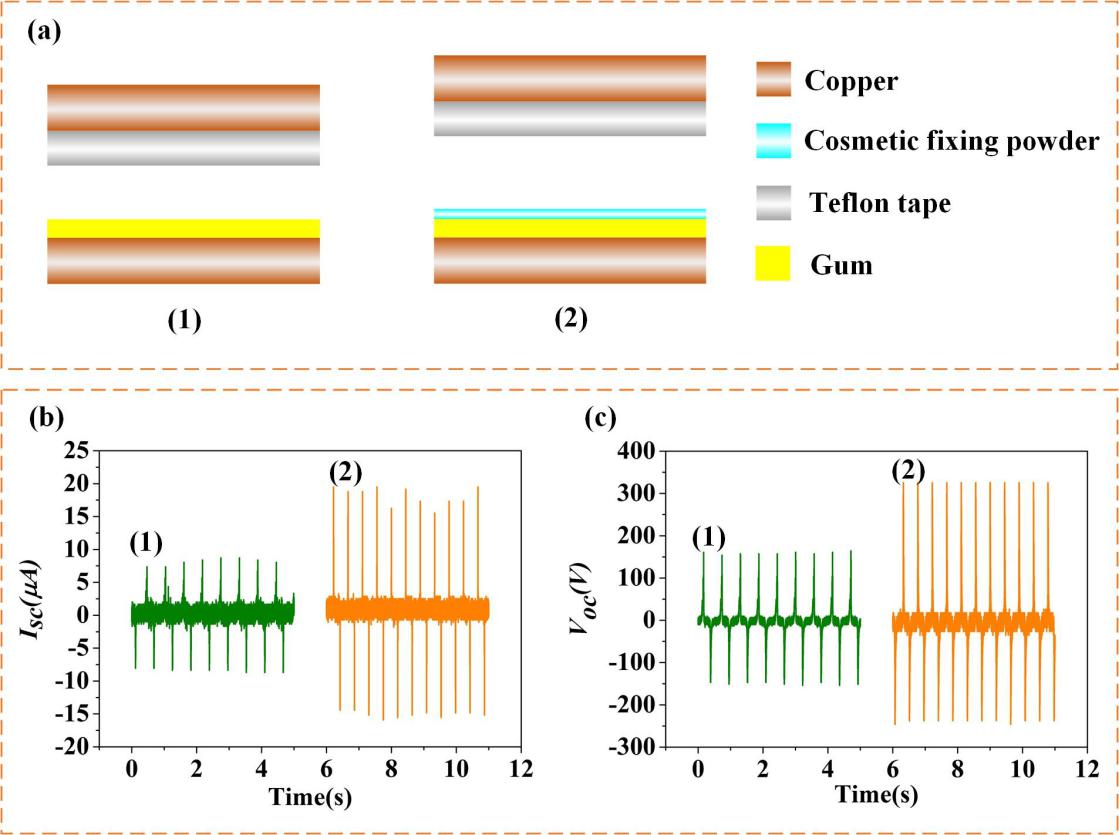
**

Fig. S1 The schematic illustration of TENG based on (a1) dried gum and (a2) cosmetic fixing powder. The (b) I_sc_ and (c) V_oc_ of gum-based TENG and CFP-TENG under the frequency of 2 Hz.

**S.2 The detailed discussions of impact of the separation displacements and frequency on the electrical performances**

As mentioned in previous work [R1], output performance of a contact-separation mode TENG is highly dependent on the contact speed. In our work, when the contact frequency increased under the same separation distance, the approximate values of I_sc_ and V_oc_ increased, and this can be attributed to the fast induction and transferring of charges under high frequency contact, as the same as previous studies of TENG [R2, R3]. In detail, if we set the amount of charge transferred between two conductive electrodes to Q during one cycle of triboelectric process, the corresponding output current (I) can be illustrated as equation (1). It is easy to understand that the higher the frequency, the greater the current generated, and the same is true for the corresponding output voltage.

$I=\frac{Q}{t}$ (1)

According to reference [R4], the PTFE (thickness: d_1_) and cosmetic fixing powder layer (thickness: d_2_) can be regarded as two dielectric layers with different permittivities. The permittivity of the PTFE, cosmetic fixing powder layer, and air are denoted as ε_1_, ε_2_, and ε_0_, respectively. The area of the friction surfaces associated with the PTFE and cosmetic fixing powder layer is S, and the distance between these surfaces is denoted as x(t)(shown in Fig. S2a). Similarly, the transferred charges induced by the potential difference are designated as Q. Therefore, the V-Q-x theoretical equation for the CFP-TENG is given as:

$V=-\frac{Q}{S\varepsilon_{0}}\left(\frac{\text{d}_{1}}{\varepsilon_{1}}+\frac{d_{2}}{\varepsilon_{2}}+x\left(t\right)\right)+\frac{\sigma x\left(t\right)}{\varepsilon_{0}}$ (2)

According to the equation (2), the output current and voltage increases continuously with the increase of separation distance, which is also demonstrated in reference [R1].


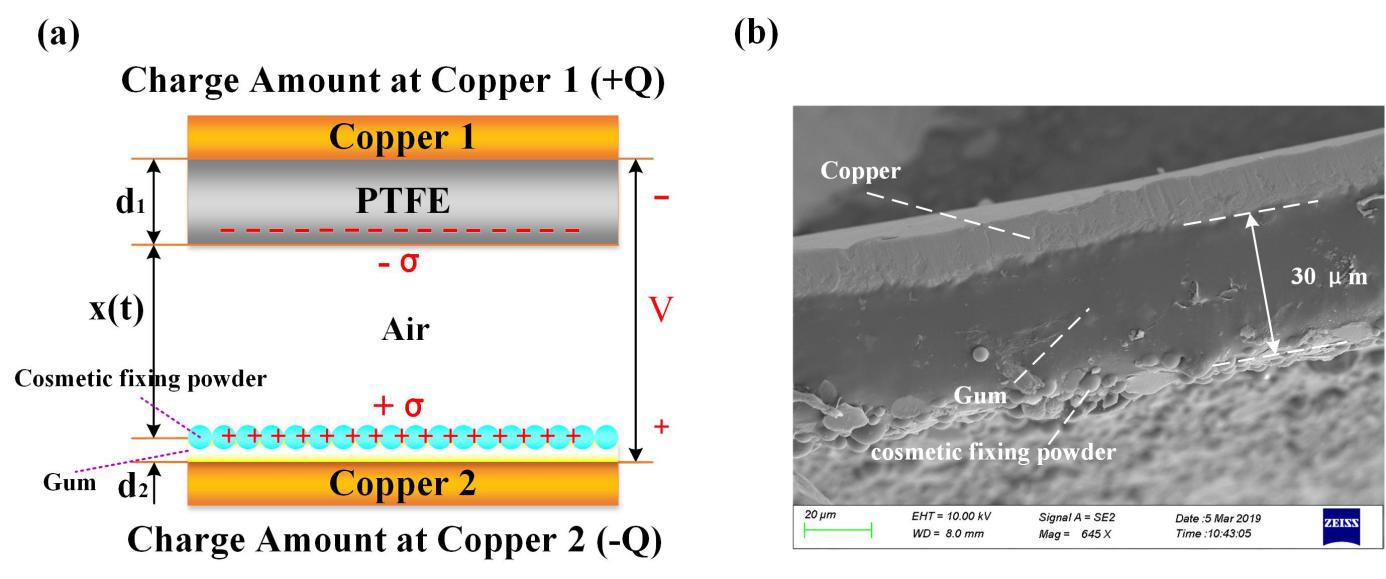


**Fig. S2** (a) Theoretical framework for dielectric-to-dielectric, attached-electrode, parallel-plate, contact-mode TENG. (b) Cross-sectional SEM image of copper/ gum/ cosmetic fixing powder layer

**Reference:**

[R1] Pan, R., Xuan, W., Chen, J., *et al*. Fully biodegradable triboelectric nanogenerators based on electrospun polylactic acid and nanostructured gelatin films. *Nano Energy*, 2018, **45**, 193-202.

[R2] Jie, Y., Jia, X., Zou, J., *et al*. Natural leaf made triboelectric nanogenerator for harvesting environmental mechanical energy. *Advanced Energy Materials*, 2018, **8**, 1703133.

[R3] Cao, R., Zhou, T., Wang, B., *et al*. Rotating-sleeve triboelectric-electromagnetic hybrid nanogenerator for high efficiency of harvesting mechanical energy.*ACS Nano*, 2017, **11**, 8370-8378.

[R4] Niu, Simiao, and Zhong Lin Wang. Theoretical systems of triboelectric nanogenerators. *Nano Energy*, 2015, **14**, 161-192.
