# Supplementary figures and images for: A triboelectric nanogenerator based on cosmetic fixing powder for mechanical energy harvesting
Source: Microsyst Nanoeng. 2019 Jul 1;5:26. doi: 10.1038/s41378-019-0066-1 (PMC6799839; doi:10.1038/s41378-019-0066-1)

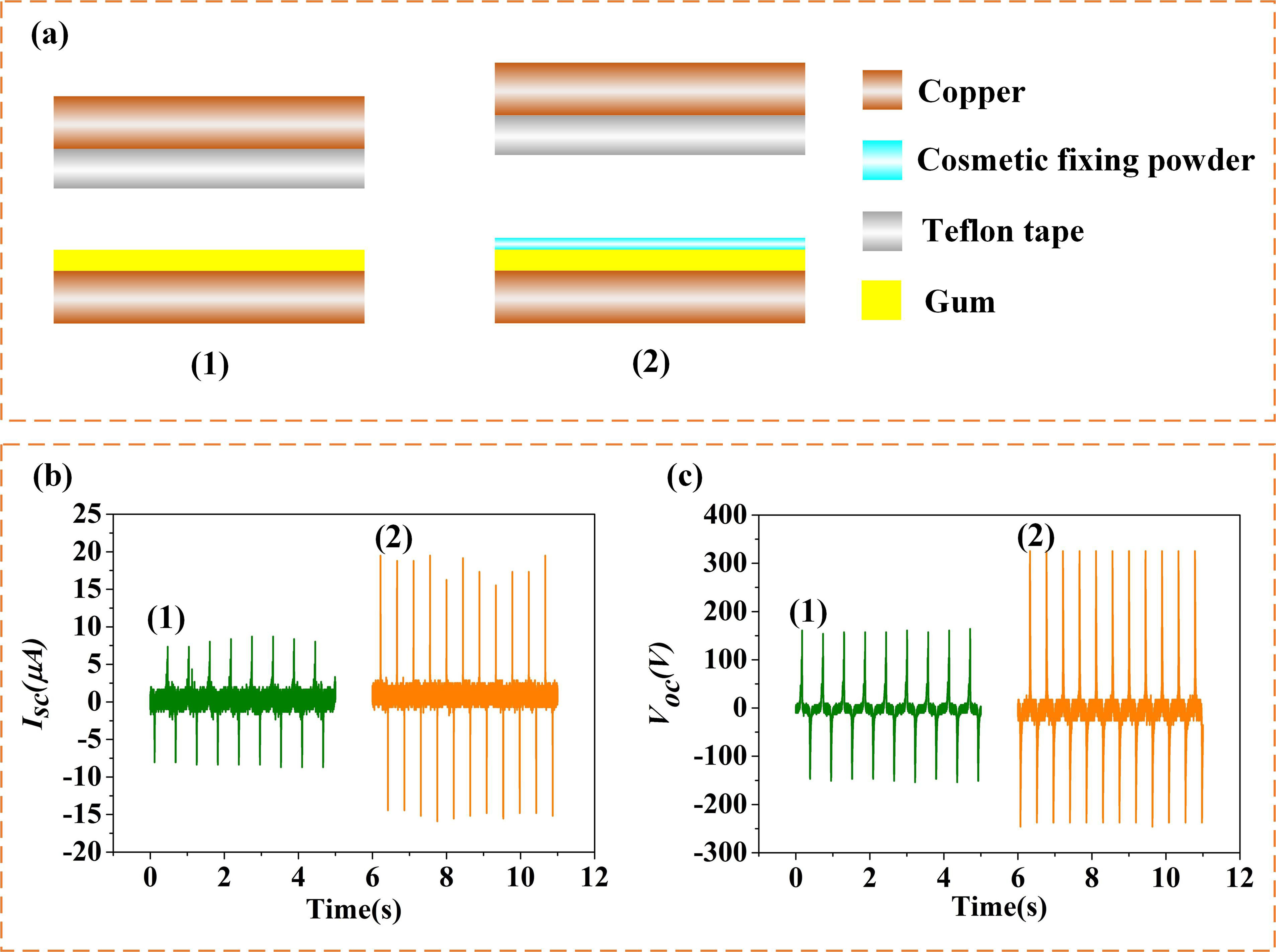

Supplement: Supplementary file 2 — Fig. S1 (supporting information) [file 41378_2019_66_MOESM2_ESM.jpg]

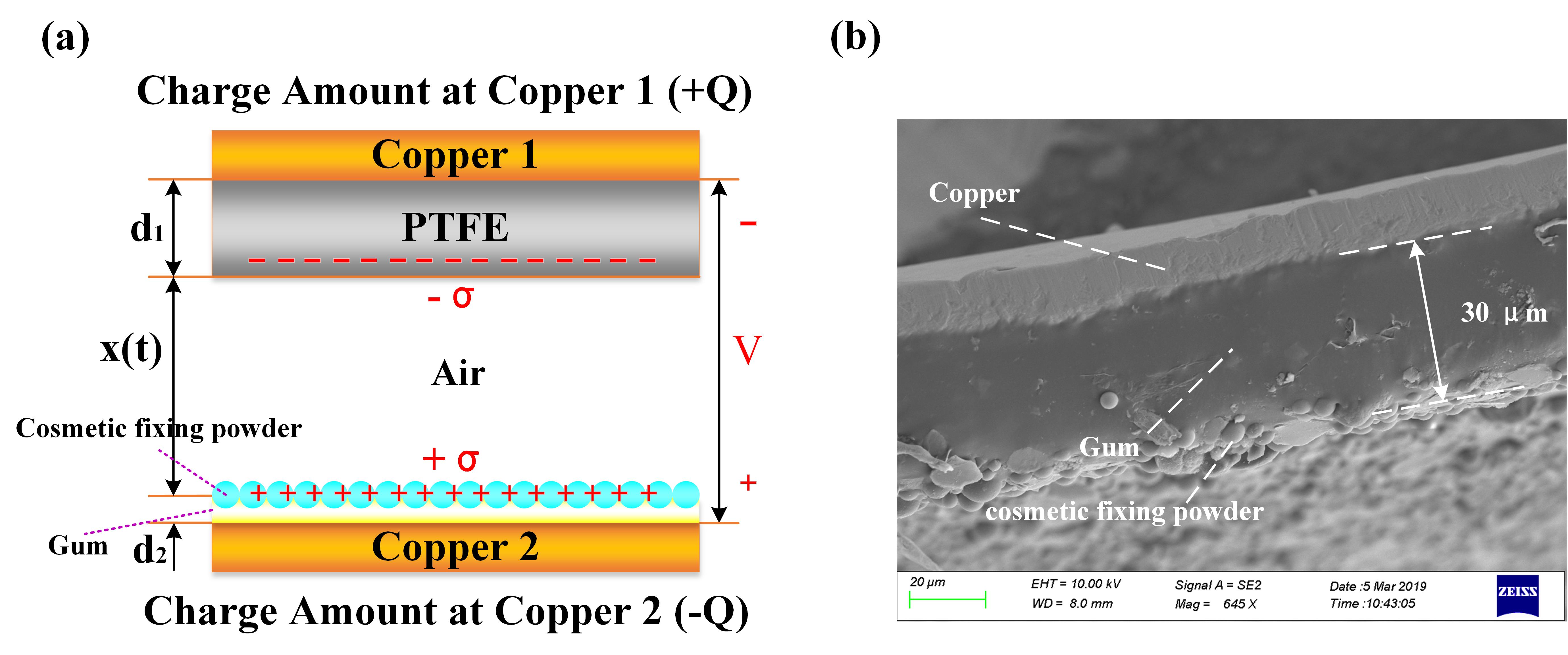

Supplement: Supplementary file 3 — Fig. S2 (supporting information) [file 41378_2019_66_MOESM3_ESM.jpg]
